# [Retracted] Stimulation of peroxisome proliferator‑activated receptor γ inhibits estrogen receptor α transcriptional activity in endometrial carcinoma cells

**DOI:** 10.3892/or.2024.8836

**Published:** 2024-11-04

**Authors:** Guiyu Zhang, Xinxin Hou, Shuhong Gao

Oncol Rep 33: 1227–1234, 2015; DOI: 10.3892/or.2015.3729

Following the publication of this paper, after having performed an independent analysis of the data shown in the various of the figures in the Editorial Office, it was identified that, in Fig. 5A and B on p. 1232 showing the results of Transwell invasion and migration experiments, the majority of the data panels exhibited overlapping sections of data, such that the affected panels, which were intended to show the results of differently performed experiments, had all apparently been derived from the same original sources.

Owing to the large number of duplications of data that have been identified in this paper, the Editor of *Oncology Reports* has decided that it should be retracted from the Journal on account of a lack of confidence in the presented data. After having been in contact with the authors, they accepted the decision to retract the paper. The Editor apologizes to the readership for any inconvenience caused.

